# Supplementary material for: Modeling glioblastoma heterogeneity as a dynamic network of cell states
Source: Mol Syst Biol. 2021 Sep 16;17(9):e10105. doi: 10.15252/msb.202010105 (PMC8444284; doi:10.15252/msb.202010105)
Supplement: Supplementary file 6 — Source Data for Figure 5 [file MSB-17-e10105-s004.zip › Figure5A_sourcedata/GSEA_3017/nosigdb_stateA.GseaPreranked.1622795026485/FELDMAN_2020_G1.html]

Details for gene set FELDMAN\_2020\_G1[GSEA]

|  || Dataset | state53017 |
| Phenotype | NoPhenotypeAvailable |
| Upregulated in class | na\_pos |
| GeneSet | FELDMAN\_2020\_G1 |
| Enrichment Score (ES) | 0.4196625 |
| Normalized Enrichment Score (NES) | 3.023183 |
| Nominal p-value | 0.0 |
| FDR q-value | 0.0 |
| FWER p-Value | 0.0 |
Table: GSEA Results Summary

  

Fig 1: Enrichment plot: FELDMAN\_2020\_G1      
 Profile of the Running ES Score & Positions of GeneSet Members on the Rank Ordered List

  

| PROBE | GENE SYMBOL | GENE\_TITLE | RANK IN GENE LIST | RANK METRIC SCORE | RUNNING ES | CORE ENRICHMENT || 1 | CDK1 |  |  | 3 | 1.006 | 0.0225 | Yes |
| 2 | MKI67 |  |  | 4 | 0.926 | 0.0462 | Yes |
| 3 | UBE2C |  |  | 7 | 0.793 | 0.0643 | Yes |
| 4 | KIF20B |  |  | 10 | 0.749 | 0.0813 | Yes |
| 5 | CEP55 |  |  | 11 | 0.747 | 0.1004 | Yes |
| 6 | BUB3 |  |  | 12 | 0.740 | 0.1194 | Yes |
| 7 | KPNA2 |  |  | 20 | 0.667 | 0.1288 | Yes |
| 8 | HIST1H4C |  |  | 21 | 0.659 | 0.1456 | Yes |
| 9 | IGFBP3 |  |  | 26 | 0.641 | 0.1576 | Yes |
| 10 | ASPM |  |  | 27 | 0.639 | 0.1740 | Yes |
| 11 | DNAJC9 |  |  | 34 | 0.615 | 0.1831 | Yes |
| 12 | CCNB1 |  |  | 37 | 0.599 | 0.1963 | Yes |
| 13 | CKS2 |  |  | 40 | 0.587 | 0.2091 | Yes |
| 14 | NCAPG |  |  | 44 | 0.556 | 0.2201 | Yes |
| 15 | CKS1B |  |  | 50 | 0.530 | 0.2281 | Yes |
| 16 | DBF4 |  |  | 55 | 0.514 | 0.2369 | Yes |
| 17 | CENPE |  |  | 62 | 0.496 | 0.2429 | Yes |
| 18 | CCNA2 |  |  | 65 | 0.495 | 0.2534 | Yes |
| 19 | TPX2 |  |  | 66 | 0.488 | 0.2659 | Yes |
| 20 | DLGAP5 |  |  | 76 | 0.475 | 0.2681 | Yes |
| 21 | HIST1H1B |  |  | 77 | 0.475 | 0.2803 | Yes |
| 22 | AURKA |  |  | 78 | 0.474 | 0.2924 | Yes |
| 23 | CDC20 |  |  | 80 | 0.472 | 0.3034 | Yes |
| 24 | VRK1 |  |  | 82 | 0.470 | 0.3144 | Yes |
| 25 | UBE2T |  |  | 83 | 0.470 | 0.3264 | Yes |
| 26 | DUT |  |  | 87 | 0.466 | 0.3350 | Yes |
| 27 | PLK1 |  |  | 89 | 0.464 | 0.3458 | Yes |
| 28 | TUBB4B |  |  | 93 | 0.462 | 0.3543 | Yes |
| 29 | CENPF |  |  | 94 | 0.461 | 0.3662 | Yes |
| 30 | TOP2A |  |  | 95 | 0.457 | 0.3779 | Yes |
| 31 | ZWINT |  |  | 106 | 0.447 | 0.3782 | Yes |
| 32 | AURKB |  |  | 108 | 0.446 | 0.3886 | Yes |
| 33 | KIF23 |  |  | 116 | 0.436 | 0.3920 | Yes |
| 34 | DEPDC1 |  |  | 119 | 0.432 | 0.4008 | Yes |
| 35 | GTSE1 |  |  | 146 | 0.411 | 0.3826 | Yes |
| 36 | CCNB2 |  |  | 147 | 0.411 | 0.3931 | Yes |
| 37 | ANLN |  |  | 152 | 0.407 | 0.3991 | Yes |
| 38 | MAD2L1 |  |  | 153 | 0.406 | 0.4095 | Yes |
| 39 | MCM7 |  |  | 171 | 0.394 | 0.4008 | Yes |
| 40 | NDC80 |  |  | 173 | 0.393 | 0.4098 | Yes |
| 41 | KIF22 |  |  | 197 | 0.377 | 0.3940 | Yes |
| 42 | CDCA3 |  |  | 203 | 0.372 | 0.3980 | Yes |
| 43 | PTMA |  |  | 206 | 0.371 | 0.4053 | Yes |
| 44 | SMC4 |  |  | 217 | 0.367 | 0.4036 | Yes |
| 45 | SPC25 |  |  | 226 | 0.363 | 0.4041 | Yes |
| 46 | CENPA |  |  | 243 | 0.352 | 0.3954 | Yes |
| 47 | H2AFZ |  |  | 245 | 0.351 | 0.4033 | Yes |
| 48 | FEN1 |  |  | 259 | 0.343 | 0.3977 | Yes |
| 49 | KNSTRN |  |  | 263 | 0.340 | 0.4031 | Yes |
| 50 | PLP1 |  |  | 272 | 0.338 | 0.4029 | Yes |
| 51 | CLSPN |  |  | 274 | 0.336 | 0.4104 | Yes |
| 52 | ESCO2 |  |  | 282 | 0.333 | 0.4111 | Yes |
| 53 | ORC6 |  |  | 283 | 0.333 | 0.4197 | Yes |
| 54 | CENPN |  |  | 310 | 0.323 | 0.3991 | No |
| 55 | HNRNPDL |  |  | 315 | 0.320 | 0.4029 | No |
| 56 | PRC1 |  |  | 350 | 0.310 | 0.3732 | No |
| 57 | ANP32B |  |  | 357 | 0.307 | 0.3744 | No |
| 58 | CDCA2 |  |  | 369 | 0.304 | 0.3700 | No |
| 59 | CKAP2 |  |  | 379 | 0.301 | 0.3678 | No |
| 60 | RPA3 |  |  | 381 | 0.300 | 0.3744 | No |
| 61 | RRM2 |  |  | 383 | 0.300 | 0.3809 | No |
| 62 | CENPW |  |  | 386 | 0.299 | 0.3864 | No |
| 63 | PHF19 |  |  | 393 | 0.297 | 0.3873 | No |
| 64 | HMGB1 |  |  | 403 | 0.292 | 0.3849 | No |
| 65 | HMGB2 |  |  | 424 | 0.288 | 0.3701 | No |
| 66 | NUSAP1 |  |  | 430 | 0.286 | 0.3719 | No |
| 67 | NUF2 |  |  | 434 | 0.285 | 0.3759 | No |
| 68 | MIS18A |  |  | 439 | 0.284 | 0.3787 | No |
| 69 | LMNB1 |  |  | 442 | 0.282 | 0.3837 | No |
| 70 | ANP32E |  |  | 453 | 0.280 | 0.3798 | No |
| 71 | ATAD2 |  |  | 470 | 0.275 | 0.3692 | No |
| 72 | KIF4A |  |  | 504 | 0.269 | 0.3395 | No |
| 73 | UBE2S |  |  | 507 | 0.269 | 0.3442 | No |
| 74 | RANBP1 |  |  | 536 | 0.263 | 0.3200 | No |
| 75 | CDKN3 |  |  | 539 | 0.263 | 0.3245 | No |
| 76 | FBXO5 |  |  | 557 | 0.258 | 0.3123 | No |
| 77 | DDX39A |  |  | 562 | 0.258 | 0.3145 | No |
| 78 | TMPO |  |  | 584 | 0.253 | 0.2977 | No |
| 79 | DLG1 |  |  | 611 | -0.257 | 0.2755 | No |
| 80 | S100B |  |  | 696 | -0.299 | 0.1901 | No |
| 81 | MALAT1 |  |  | 702 | -0.302 | 0.1923 | No |
| 82 | SCG2 |  |  | 907 | -0.564 | -0.0191 | No |
| 83 | CTGF |  |  | 976 | -1.182 | -0.0641 | No |
| 84 | CYR61 |  |  | 978 | -1.296 | -0.0320 | No |
| 85 | IGFBP5 |  |  | 983 | -1.595 | 0.0044 | No |
Table: GSEA details [plain text format]

  

Fig 2: FELDMAN\_2020\_G1: Random ES distribution      
 Gene set null distribution of ES for **FELDMAN\_2020\_G1**
